# Classifying Advanced Driver Assistance System (ADAS) Activation from Multimodal Driving Data: A Real-World Study

**DOI:** 10.3390/s25196139

**Published:** 2025-10-04

**Authors:** Gihun Lee, Kahyun Lee, Jong-Uk Hou

**Affiliations:** Department of Computer Engineering, Hallym University, Chuncheon 24252, Republic of Korea

**Keywords:** advanceddriver assistance systems, multimodal deep learning, vehicle forensics, IMU sensors

## Abstract

Identifying the activation status of advanced driver assistance systems (ADAS) in real-world driving environments is crucial for safety, responsibility attribution, and accident forensics. Unlike prior studies that primarily rely on simulation-based settings or unsynchronized data, we collected a multimodal dataset comprising synchronized controller area network (CAN)-bus and smartphone-based inertial measurement unit (IMU) signals from drivers on consistent highway sections under both ADAS-enabled and manual modes. Using these data, we developed lightweight classification pipelines based on statistical and deep learning approaches to explore the feasibility of distinguishing ADAS operation. Our analyses revealed systematic behavioral differences between modes, particularly in speed regulation and steering stability, highlighting how ADAS reduces steering variability and stabilizes speed control. Although classification accuracy was moderate, this study provides one of the first data-driven demonstrations of ADAS status detection under naturalistic conditions. Beyond classification, the released dataset enables systematic behavioral analysis and offers a valuable resource for advancing research on driver monitoring, adaptive ADAS algorithms, and accident forensics.

## 1. Introduction

Advanced driver assistance systems (ADAS) [1] have emerged as a key technology for improving road safety. With the imminent commercialization of autonomous driving technologies, the ability to accurately identify the activation status of ADAS is no longer a theoretical issue but a practical necessity for transportation safety and accident forensics with legal ramifications. Distinguishing between automated and manual driving modes plays a critical role in clarifying driver versus system control, which directly affects insurance claims, liability determination, and the design of regulatory frameworks.

However, most existing studies have relied on simulation environments or controlled laboratory experiments, limiting their applicability to naturalistic driving. Maag et al. [2] reported a reduction in emergency braking frequency when ADAS was engaged. Biondi et al. [3] highlighted the behavioral trade-offs associated with warning systems. Morando et al. [4] investigated specific automation functions, yet their findings lack generalizability to uncontrolled real-world conditions. In particular, the absence of synchronized multimodal data collected from real vehicles under consistent conditions has critically constrained systematic analysis of ADAS activation and its behavioral implications.

Unlike fully automated driving systems (FDS), ADAS provides only partial assistance with limited perception and control capabilities, requiring continuous driver supervision [5]. This partial automation not only complicates behavioral modeling but also creates scenarios where system disengagement or driver overtrust can lead to accidents. Consequently, there is an urgent need for real-world behavioral datasets that reflect naturalistic ADAS usage and robust classification methods capable of distinguishing ADAS and manual modes under realistic conditions.

Accurate identification of ADAS activation can clarify the timing of driver versus system intervention in the event of an accident, facilitate evidence-based investigations, and streamline insurance processes. Traditional in-vehicle recording devices such as event data recorders (EDRs) have been widely adopted for accident forensics, but their logged variables are typically limited to coarse-grained events (e.g., airbag deployment, crash pulses, basic speed traces) and often lack information on the nuanced behavioral dynamics that characterize ADAS usage. Moreover, EDR data are not standardized across manufacturers, and critical indications for distinguishing between manual and assisted driving modes are frequently unavailable. Beyond safety and forensics, ADAS mode detection grounded in real driver behavior can advance adaptive warning and control strategies, enabling personalized driver support services that integrate fatigue and attention monitoring.

To address these gaps, this study collected synchronized inertial measurement unit (IMU) sensor and controller area network (CAN)-bus signals from the same drivers on a consistent highway section under both ADAS-on and manual driving modes. Using this dataset, we developed a session-level behavioral prediction framework and performed an in-depth statistical analysis of driver behavior. The main contributions are as follows:

- Behavior-based driving mode classification: A machine learning-based model was developed using CAN-bus and IMU features, demonstrating that driver behavior alone can reliably indicate ADAS engagement.
- Quantitative analysis of ADAS-induced behavioral changes: Consistent differences in steering variability, speed regulation, and torque behavior between ADAS and manual driving were identified.
- Construction of a real-world multimodal dataset: A synchronized IMU and CAN-bus dataset was established by collecting under controlled yet naturalistic conditions, providing a valuable resource for future research.

In summary, this study establishes a real-world multimodal framework and systematically compares driver behavior under consistent conditions, thereby providing a foundation for ADAS-related behavioral modeling, accident forensics, and the design of adaptive driver support systems.

## 2. Background

### 2.1. Definition of Advanced Driver Assistance Systems (ADAS)

The automotive industry has pursued stepwise automation since the 1990s, beginning with rear parking sensors and anti-lock braking systems (ABS). Currently, according to the Society of Automotive Engineers (SAE) J3016 standard, driving automation is categorized into levels 0 to 5, further classifying Level 1 (driver assistance) and Level 2 (partial automation) as ADAS. ADAS functions can be grouped into three categories: (i) providing information (warnings and alerts), (ii) active intervention (steering and braking), and (iii) authority sharing and transfer. Each category differs in allocation of sensing, decision-making, and control responsibilities between the driver and system. Recent research argues that purely functional categories are insufficient to explain safety performance; thus, a three-dimensional framework based on information, task demand, and control authority was proposed [6].

Large-scale crash statistics from real-world driving [7] indicate that among 7.7 million vehicles manufactured between 2015 and 2023, ADAS-equipped cars experienced on average a 27% reduction in forward-collision crashes and an 11% reduction in lane-departure crashes. Both the European Union (EU) and the United States have incorporated ADAS availability into the New Car Assessment Program (NCAP), accelerating the widespread adoption of ADAS technologies across the industry.

However, in SAE Level 2 (partial automation), driver overtrust has been reported, whereby drivers neglect vigilance by over-relying on the system [8]. In an on-road experiment conducted by the Insurance Institute for Highway Safety (IIHS) [9], applying shared control reduced hands-off time by approximately 40%. However, performance degradation in adverse weather, lane-detection failures, and sensor false positives or missed detections remain unresolved challenges [10]. These issues underscore the need for complementary approaches that combine advanced sensing and algorithmic techniques.

Against this background, the representative ADAS core functions widely used in real-road environments are summarized below.

- FCW & AEB: Forward collision warning and autonomous emergency braking using camera- and radar-based risk prediction.
- ACC/SCC: Adaptive or smart cruise control for longitudinal distance keeping.
- LKA/LFA: Lane keeping assist and lane following assist for lateral steering control.
- BSD & RCTA: Blind-spot detection and rear cross-traffic alert for lateral and reversing support.
- HDA: Highway driving assist integrating SCC and LFA to provide simultaneous longitudinal and lateral control.

From a product perspective, major manufacturers such as Hyundai, Mercedes-Benz, GM, Ford, Nissan, and Toyota offer broadly similar ADAS building blocks centered on longitudinal distance keeping (ACC/DRCC), lateral lane centering (LKA/LFA), and optional automated lane changes. Real-world behaviors differ in sensing suites, dependence on mapping, and human–machine interface (HMI) strategies; however, they generally converge to a common operational pattern of “distance keeping + lane centering + (optional) lane change,” enabling cross-brand comparisons and analysis.

This study focuses on ADAS functions that play a critical role in real-world driving and adopts a platform that broadly encompasses the core capabilities commonly provided across manufacturers. Data collection was performed using a Tesla Model 3, employing (1) highway driving assistance to integrate SCC and LFA (hereafter, HDA) and (2) automatic lane change (ALC) to trigger a maneuver when the turn-signal stalk is activated, thereby executing it safely displaying situational awareness of surrounding traffic. We selected Tesla because its feature set spans the common ADAS axes consisting of longitudinal control, lateral control, and lane-change execution and provide clear event boundaries (e.g., turn-signal activation) that facilitate labeling and annotation. In addition, Tesla incorporates a wide range of ADAS features and continuously improves the algorithms and control policies via over-the-air (OTA) software updates. This makes it well suited for tracking performance changes over time within a consistent platform and for analyses that reflect the latest functionality. In particular, HDA and ALC are key modules that simultaneously manage longitudinal and lateral control while supporting driving decisions, thereby revealing the essence of ADAS in terms of sensor fusion, real-time control, and transitions in driver–system authority.

### 2.2. Related Work

The influence of ADAS on driver behavior was first extensively investigated through simulator-based studies [2,3,11]. These works quantitatively analyze how functions such as auditory alerts or lane departure warnings affect driver acceleration, braking, and steering patterns. However, simulators are limited in reflecting the real-world road environment complexity, which restricts the generalizability of their findings. To overcome these limitations, the research paradigm is expanding toward using naturalistic driving data to acquire realistic insights into how drivers use and interact with ADAS in real-world environments [12,13]. In particular, research on partial automation (Level 2) has reported that drivers spend more time looking away from the road or exhibit altered gaze and steering patterns during system interventions [4,14,15].

The field has grappled with the trade-off between the experimental control of simulators and ecological validity of naturalistic data. To bridge this gap, an emerging hybrid paradigm seeks to combine the controllability and repeatability of simulations with the realism of physical vehicle testing. A prime example is the vehicle-in-virtual-environment (VVE), a methodology where the physical dynamics of steering, acceleration, and braking in a real vehicle are synchronized and reflected in real time within a virtual environment [16]. This allows the testing of specific high-risk scenarios without physical danger. The rise of hybrid paradigms such as VVE underscores the importance of naturalistic data collection in our study. While VVE evaluates system responses to pre-defined scenarios, our approach is essential for discovering driver behavior patterns and system interactions during unscripted, everyday driving situations.

The evolution of research goals has also driven a shift in data collection paradigms. Early efforts focused on large-scale datasets for general-purpose autonomous vehicle perception and prediction, such as the Honda Research Institute driving dataset (HDD) [17]. Despite being valuable, HDD was not collected with explicit control over ADAS ON/OFF states, limiting its utility for direct behavioral comparisons between these two modes. In contrast, recent multimodal driver-monitoring datasets such as manD 1.0 demonstrate the benefits of fusing heterogeneous sensors, including gaze and biosignals. However, they are often collected using static simulators, thereby lacking real-world vehicle dynamics [18]. To overcome these limitations, a new trend is the creation of specialized, purpose-driven multimodal datasets. For instance, datasets are now being built to evaluate specific ADAS functions such as lane keeping assist [19] or to analyze driver behavior during secondary tasks in Level 3 automation [20]. Our dataset contributes to this trend by providing synchronized CAN-bus and IMU data collected under controlled naturalistic conditions, explicitly controlling the ADAS ON/OFF state for the same drivers across identical routes, which enables a focused analysis of the behavioral impact.

Analyzing vehicle states with CAN-bus and IMU sensors is not a new approach; however, its application has varied significantly based on the research objective. Many existing studies using CAN or physiological data focus on detecting anomalous or hazardous driving events [21,22], formulating the problem as one of outlier detection, which is fundamentally different from the goal of classifying subtle pattern differences between two normal operating modes (manual vs. ADAS). Similarly, these time-series data are widely used for in-vehicle intrusion detection systems (IDS), which aim to identify malicious hacking attacks [23]. The signals from such extreme events are markedly different from the nuanced behavioral shifts between two legitimate driving modes. Research that is more directly related to our work has attempted to classify specific driving contexts, such as scenarios (e.g., cornering, lane changes) [24], demonstrating the feasibility of classifying vehicle states from sensor data by focusing on the vehicle’s external circumstances rather than its internal system state. Furthermore, driver-adaptive systems that learn individual preferences show the potential to extract high-level information from vehicle sensors [25,26]. The ability to identify individual drivers from CAN-bus data alone proves that unique driving habits leave a “fingerprint” in the data [27]. Such fine-grained differences between individuals if learned, would strongly suggest that the more systematic and consistent control patterns of an ADAS are also distinguishable. These studies show a progression in vehicle data analysis from low-level state estimation (e.g., speed) to high-level context classification (e.g., driver ID, ADAS mode). Our research directly addresses the high-level task of ADAS mode classification, which requires machine learning approaches capable of learning the subtle, distributed patterns that differentiate manual driving from system-assisted control.

In summary, prior research has largely focused on simulator-based experiments, vision-based detection, general anomaly analysis, or evaluations of individual functions. However, few studies have systematically compared ADAS ON/OFF conditions using the same drivers on the same route with synchronized CAN and IMU data. This study aims to address this gap.

## 3. Collecting the Dataset

We present a real-world multimodal driving dataset, which was collected to analyze the influence of ADAS operations on driver behavior. The dataset includes synchronized CAN-bus and IMU sensor data, recorded as four drivers drove along the same highway route under both ADAS and manual driving modes. This section outlines the acquisition protocol and key characteristics of the dataset, which was designed to enable systematic analysis of driver behavior under controlled yet naturalistic driving conditions.

### 3.1. Data Collection Protocol

To investigate the relationship between ADAS activation and driver behavior, we constructed a multimodal naturalistic driving dataset. The data were collected using a 2022 Tesla Model 3 equipped with synchronized sensors. Four drivers (three males and one female) participated in the study. Each driver completed two highway routes: from Chuncheon to Hongcheon (ctoh) and from Hongcheon to Chuncheon (htoc). Each route was driven twice, once in manual mode (Drive) and once with ADAS-enabled, including lane-keeping and adaptive speed control. This design ensured controlled comparisons by maintaining consistent environmental and route conditions across modes.

**Figure 1:**
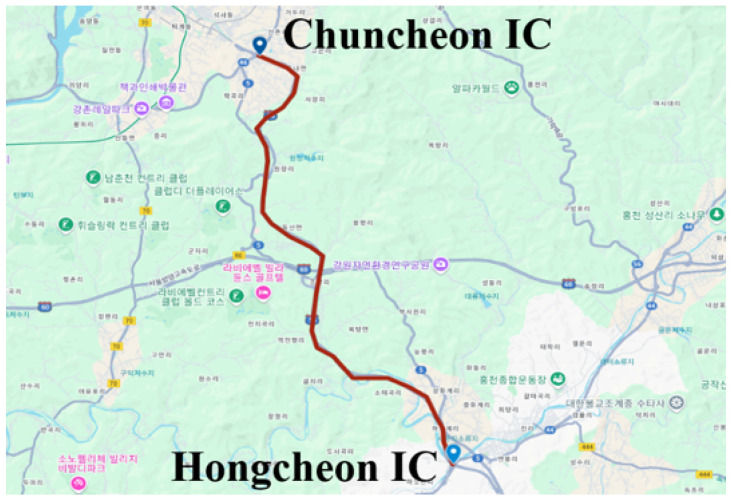
Data collection route: The highway section between Chuncheon IC and Hongcheon IC. (ctoh/htoc).

Figure 1 illustrates the highway routes used for data collection. All sessions were conducted during daytime under normal weather conditions. Special conditions such as rain, snow, or nighttime driving were excluded to maintain sensor consistency while preserving a naturalistic driving environment.

### 3.2. Dataset Details

The dataset consists of two synchronized modalities: (1) in-vehicle CAN-bus data containing vehicle dynamics information and (2) IMU sensor data capturing driver movement and vehicle trajectory. Each modality is accompanied by metadata including a timestamp, driver identifier (driver ID), driving mode (manual or ADAS), route (ctoh or htoc), and round number (1 or 2). All modalities are time-aligned at fixed sampling rates according to sensor specifications.

IMU Data (Inertial Measurement Unit) The IMU data were collected using the built-in sensors of a Samsung Galaxy A24 smartphone, which provided accelerometer, gyroscope, and magnetometer measurements. The smartphone was mounted on the vehicle dashboard, and a custom logging application (APK) was used to record the IMU data in real time. Accelerometer and gyroscope signals were sampled at 100 Hz, whereas magnetometer data were sampled at 50 Hz, as required. By leveraging a commercial smartphone, IMU data could be obtained in a cost-efficient manner while ensuring high reproducibility, thereby eliminating the need for additional external sensor installations. The IMU provides signals along three orthogonal axes (x, y, z). In vehicle dynamics, these correspond to roll (rotation around the longitudinal x-axis, causing side-to-side tilting), pitch (rotation around the lateral y-axis, causing nose up-and-down motion), and yaw (rotation around the vertical z-axis, causing left-right turning).

CAN-bus Data The CAN-bus data were extracted from the in-cabin CAN network of the Tesla vehicle using an Kvaser Leaf Light v2 interface module. The module was connected to the vehicle’s on-board diagnostics (OBD)-II port and directly linked to a laptop (or an embedded personal computer (PC)) via universal serial bus (USB) for real-time frame collection. Signal decoding was performed using the “tesla_can.dbc” file, which specifies message structures, signal names, bit lengths, scaling factors, offsets, and minimum/maximum values for each CAN ID.

Driving Environment Experiments were conducted on two highway segments: Route A (Chuncheon IC → Hongcheon IC) and Route B (Hongcheon IC → Chuncheon IC). Both segments are typical two- to three-lane highways with a lane width of approximately 3.5 m, well-maintained pavement, and stable surface friction, providing reliable driving conditions. All experiments were conducted on clear days (no precipitation, good visibility) between 2 p.m. and 6 p.m., a period chosen to minimize traffic density variations while ensuring a naturalistic driving environment.

ADAS Operation Settings The test vehicle was equipped with an ADAS system, including adaptive cruise control (speed maintenance) and lane keeping assist. Data collection in ADAS-on mode began at highway entry ramps, where ADAS was switched from off to on and ended at exit ramps, when ADAS was switched off. For manual driving, data were recorded along the same routes with ADAS fully disabled. Each transition of ADAS activation was visually indicated to the driver by dashboard light-emitting diode (LED) signals. In ADAS-on mode, automatic steering support and lane centering functions reduced the driver’s direct control burden and altered the acceleration and braking patterns. To capture these effects, synchronized dashcam, IMU, and CAN-bus data were collected in both ADAS on and off conditions, enabling comparative analyses of behavioral changes before and after mode transition.

**Figure 2:**
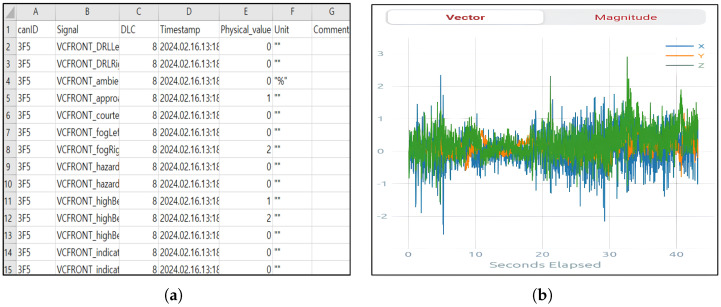
Collected data samples: (**a**) CAN-bus data frames collected via the OBD-II port, containing vehicle dynamics such as motor torque. (**b**) Example of 3-axis IMU (accelerometer) signals measured by a smartphone.

Table 1 summarizes the main features used in this study, including vehicle speed, steering angle, motor torque, and regenerative braking status. Speed- and steering-related features were particularly identified as critical for understanding control transitions under ADAS and served as essential inputs for behavioral modeling. Representative samples from each modality are shown in Figure 2.

## 4. Methodology

**Figure 3:**
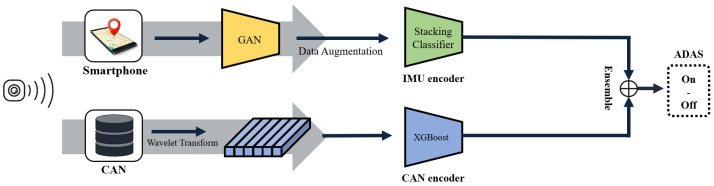
Proposed ADAS ON/OFF prediction framework. Two data streams, smartphone IMU and vehicle CAN-bus, are processed independently through their respective encoders. The prediction probabilities from each classifier are then ensembled for the final ADAS mode determination.

The ADAS activation (ON) vs. manual driving (OFF) was classified by independently modeling CAN-bus and IMU modalities and subsequently fusing their prediction probabilities (Figure 3). For each modality, we performed preprocessing, windowing, feature extraction, and classifier training. The final ON/OFF decision was obtained by combining the two posteriors. Performance was evaluated using four-fold cross-validation (route × round). We report the fold-wise mean and dispersion.

### 4.1. Feature Extraction and Prediction Pipeline Using CAN-Bus Data

The ADAS activation was detected from 1 Hz CAN-bus data via: (1) signal selection & normalization, (2) windowing, (3) wavelet features, and (4) window-level prediction with calibration. The design objective was low computational cost while preserving time–frequency cues reflecting driver actions.

Among the candidate channels in Table 1, we kept driving-dynamics signals (accelerator/brake, speed, steering, torque) and removed identifiers/metadata (IDs, cyclic redundancy check (CRC), counters), near-monotonic channels (odometer-like), and channels with class-imbalanced presence. To prevent leakage, we also dropped session separators with session-level median that almost perfectly separated ADAS/Drive on the training split (ROC–AUC ≥0.995) and backfilled with the next-best candidates. All retained channels were scaled by *global* train-time min–max constants, mapping NaN to 0.

Signals were pivoted to 1 Hz and segmented with a sliding window of *L* seconds and stride Δ:

(1) $$W_{k}=\left\{x_{t}|t\in \left[k,k+L\right)\right\},\;L=120,\;\Delta =1\;\left(\mathrm{speed}\text{-}\mathrm{up}\text{:}\;\Delta =5\right).$$

To capture short-term dynamics, we appended first- and second-order temporal differences per channel. The window length of L=120 s was chosen following the work of Kwak et al. [28], to effectively capture stable driving patterns, balancing statistical stability of driver behavior with sensitivity to ADAS transitions. A 1 s stride provides dense coverage without excessive redundancy.

For each channel in a window (including derivatives), we computed a one-dimensional discrete wavelet transform (1D DWT) (db4) to a level determined by *L* and data length. Specifically, we used Level 2 for L=60 s, Level 3 for L=90 s, and Level 4 for L∈{120,150} s (capped by the signal’s maximal admissible level). Lettng $\left\{C_{j}\right\}$ be the scale-wise coefficients, we extracted

(2) $$E_{j}=\sum_{k}{\left|C_{j}\left(k\right)\right|}^{2},\;p_{j}=\frac{E_{j}}{\sum_{\ell }E_{\ell }},\;\mathrm{WEE}=-\sum_{j}p_{j}\ln p_{j},$$

and added histogram-based Shannon entropy and summary statistics for raw/cA/cD (percentiles 5/25/75/95, mean, median, variance, std, RMS, skew, kurtosis). This design is motivated by prior driver modeling studies. Kwak et al. [28] showed that wavelet-based energy and entropy descriptors of driving signals can effectively capture driver-specific control patterns and significantly improve driver identification accuracy when combined with machine learning classifiers such as extreme gradient boosting (XGBoost). Their findings suggest that wavelet features emphasize meso-scale fluctuations in steering and speed signals that are often invisible to raw statistics. Adopting this rationale, we employed wavelet + statistical summary features to capture subtle behavioral differences between ADAS ON and manual driving conditions while maintaining low computational cost.

A probabilistic binary classifier was trained on window features. XGBoost was chosen for its robustness on tabular statistical features, low training/inference cost, and interpretability of feature importance. For the operating threshold, training sessions were split into train_inner and calib; the model was fit on train_inner, and the threshold was chosen on calib session-level scores via Youden’s *J* (maximizing TPR–FPR). At test time, window-level probabilities were output by the model; session aggregation and cross-modal fusion were handled by the Voting Strategy and Decision Fusion modules. Four-fold splits were used by route and round (ctoh-0/1, htoc-0/1), holding out entire sessions.

### 4.2. Feature Extraction and Prediction Pipeline Based on IMU Data

The ADAS activation was predicted from 6-axis smartphone IMU (accelerometer, gyroscope) signals via: (1) signal merging & normalization, (2) sliding-window feature extraction, and (3) lightweight classifier training. This pipeline outputs clip-level probabilities for downstream aggregation and fusion.

Accelerometer and gyroscope were aligned by nearest neighbor (tolerance 0.05 s), merged, and resampled to 2 Hz. Sessions were defined by driver–mode–route–round. Following the methodology of Ahmadian et al. [29], we adapted the window size for our data. Given that our dataset’s session lengths are shorter than those in the reference study, we conducted preliminary experiments and ultimately adopted 5-min (L=300 s) windows with 80% overlap (approximately 60 s stride):

(3) $$W_{k}=\left\{x_{t}|t\in \left[k,k+L\right)\right\},\;L=300\;s,\;\Delta =60\;s,$$

where $x_{t}\in R^{6}$ is the 6-axis vector. Each window is one input clip, and session ID, segment index, and start time for aggregation are retained.

By default, features were 100-bin histograms per axis over [μ−2σ,μ+2σ] (session-level μ,σ), concatenated across six axes. As an alternative, TSFEL descriptors (time/statistical/spectral) can be used. Standardization used a StandardScaler fit on training data only; features with train-set correlation >0.95 were removed, followed by row-wise $\ell_{2}$ normalization. This discrete-feature, ensemble modeling setup was motivated by prior IMU-based driver studies [29] and extended here to ADAS detection.

The classifier was a stacking ensemble consisting of linear support vector classification (LinearSVC), multilayer perceptron (MLP), k-nearest neighbor (KNN), random forest as base learners and logistic regression as meta-learner to generate clip-level posteriors. This ensemble design leverages complementary decision boundaries from multiple lightweight learners, which has been proven effective in prior IMU-based driver studies [29].

As an exploratory component, we evaluated conditional generative adversarial network (GAN)–based augmentation by applying a one-level DWT (db2) to obtain per-axis approximation/detail channels (12 channels in total), synthesizing windows using a deep convolutional GAN (DCGAN) [30] and reconstructing time-domain signals via inverse DWT (IDWT). A previous study [29] reported the benefits of GAN-based augmentation in related sensor or time-series tasks; however, in our setting this approach did not yield consistent improvements and tended to increase cross-fold variance. For example, compared to the non-augmented configuration (Accuracy ≈ 0.792, F1 ≈ 0.822), the augmented variant showed slightly lower mean performance (Accuracy ≈ 0.783, F1 ≈ 0.785). Accordingly, all the final results in this paper are reported without GAN augmentation. The remaining evaluations follow the same four-fold split by route and round, with clip-level posteriors passed to the Voting Strategy and Decision Fusion modules.

### 4.3. Decision Fusion

The final ADAS ON/OFF decision is obtained by combining the posterior probabilities estimated from the CAN-bus and IMU classifiers. Let $p^{CAN}\left(y=1|W_{k}\right)$ and $p^{IMU}\left(y=1|S_{k}\right)$ denote the probability of ADAS being active given the CAN-bus window $W_{k}$ and IMU segment $S_{k}$, respectively. The fused probability is defined as a convex combination:

(4) $$p\left(y=1|W_{k},S_{k}\right)=\alpha \;p^{CAN}\left(y=1|W_{k}\right)+\left(1-\alpha \right)\;p^{IMU}\left(y=1|S_{k}\right),$$

where 0≤α≤1 controls the relative weight between the two modalities. The final decision is:

(5) $${\hat{y}}_{k}=\arg\max_{y\in \{0,1\}}\;p\left(y|W_{k},S_{k}\right).$$

In this study, α was set to 0.5, assigning equal weight to CAN-bus and IMU classifiers. It represents a conservative approach that assumes equal informational value from both modalities. This equal weighting also prevents overfitting to a single data source. Final classification performance was further enhanced using a majority voting strategy that aggregates predictions across multiple sensor clips (e.g., three, ten, and all) within a session.

Although deep sequence models such as long short-term memory (LSTM) and convolutional neural networks (CNNs) have been widely applied to time-series classification, we deliberately opted for lightweight ensemble and tree-based classifiers. The primary reason was dataset size: our multimodal collection comprises only four drivers and several highway runs, which is insufficient for robustly training high-capacity neural architectures without severe overfitting [31]. In addition, our design target emphasizes computational efficiency and interpretability for potential in-vehicle deployment. Compared with deep learning models, ensemble and gradient-boosted trees achieve competitive accuracy on feature-engineered representations while offering fast inference and clearer feature contributions, which is consistent with the findings that tree-based models often outperform deep networks on tabular data [32].

## 5. Experiments

This section describes the experimental design for distinguishing between ADAS activation (ON) and manual driving (OFF) using CAN-bus and IMU modalities collected during real highway driving. We first evaluated ADAS classification performance based on the statistical features derived from CAN-bus data and then applied the same cross-validation procedure to the IMU-based approach. Subsequently, we report the final classification performance of each modality. In addition, an independent-sample *t*-test was conducted on 22 key driving features (e.g., speed, steering, torque), and boxplot visualizations were employed to analyze the statistical and intuitive behavioral changes induced by ADAS. Combined, these experiments validate modality-specific and multimodal ADAS detection accuracy while quantitatively identifying behavioral changes caused by automation, thereby providing practical evidence for behavior-based driver monitoring and accident forensic systems.

### 5.1. Comparison Baselines

For fair benchmarking, we reproduced four independent baselines (two CAN-based and two IMU-based) under the same preprocessing, normalization, sliding-window, and session-level aggregation used in our pipeline. All methods were evaluated using identical four-fold (route × round) cross-validation. Kwak et al. [27] standardized the core window-level statistics (mean/median/std) and trained a random forest (100 trees, depth 5), using the same folds. We obtained session-level scores by averaging window softmax probabilities and report Accuracy/Precision/Recall/F1. Khan et al. [33] constructed OBD-II CAN statistical features using 120-s windows with global min–max normalization and performed window-level prediction by automatically selecting among kNN, SVM, logistic regression, and decision tree via GroupKFold area under the curve (AUC). Window outputs were summarized at the session level with fixed voting, and the decision threshold was adjusted using Youden’s *J*. Sánchez et al. [34] converted 1D acceleration to 2D representations (spectrogram/feature maps) and classified using a ResNet-50 backbone and gated recurrent unit (GRU) head. A transform-then-classify recipe was retained, and window outputs were stabilized via fixed-size bundle aggregation, reporting Accuracy/Precision/Recall/F1. Tanprasert et al. [35] used 7-s windows with 2-s hop to summarize acceleration dynamics (e.g., $a_{\mathrm{dyn}}$, jerk) and global positioning system (GPS) speed features, applied standardization, and employed an MLP classifier with optional one-class SVM (OCSVM)-based soft gating. The training folds were split into inner/calibration subsets to tune window and session thresholds via Youden’s *J* before retraining on all training data and evaluating on the held-out fold.

### 5.2. Evaluation Procedure for ADAS ON/OFF Prediction

To evaluate the generalization performance of the ADAS mode classification models, we designed a four-fold cross-validation scheme based on route direction (ctoh/htoc) and session round (1/2). Each fold corresponds to a complete driving session (including both ADAS and manual modes) performed by both drivers (Driver A and Driver B):

- Fold 1: ctoh, Round 1, Drivers (A, B)
- Fold 2: ctoh, Round 2, Drivers (A, B)
- Fold 3: htoc, Round 1, Drivers (A, B)
- Fold 4: htoc, Round 2, Drivers (A, B)

In each experiment, one fold was used as the test set, whereas the remaining three folds were used as the training set. The final performance is reported as the average across the four folds. No separate test set was used; instead, four-fold cross-validation was adopted because of the limited data size (two rounds × two routes × two drivers). Since each session presents distinct conditions (route and round), leveraging all the available data for evaluation is critical for ensuring the robustness of the model across different scenarios.

This validation strategy offers the following advantages: (1) each test fold contains a complete driving session, enabling evaluation of session-level generalization capability; (2) temporal and situational separation minimizes the risk of data leakage; and (3) despite the limited dataset size, the framework provides a rigorous test of adaptability to previously unseen routes and conditions.

### 5.3. Statistical Analysis of Driving Behavior

To quantitatively compare the impact of ADAS mode on driver behavior, we conducted a statistical analysis using 21 features derived from CAN-bus signals (C1–C7) and nine features derived from IMU signals (S1), as summarized in Table 1. Each feature corresponds to summary statistics (mean, standard deviation, median) extracted from key driving signals such as speed, steering angle, and torque, as well as axis-specific measurements.

An independent sample *t*-test was performed to compare the distribution of each feature between ADAS and manual (Drive) modes. The analysis was restricted to clearly segmented driving sessions with distinct ADAS and Drive conditions. Features with a *p*-value less than 0.05 were considered to exhibit statistically significant differences.

In addition, the mean values of each feature across the two modes were compared to identify the direction of behavioral change, i.e., whether a given behavior increased or decreased under ADAS activation.

**Figure 4:**
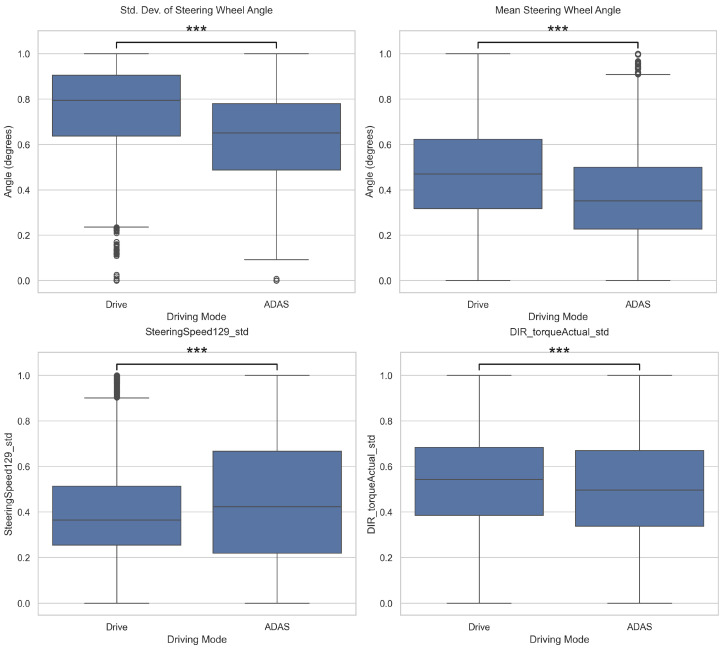
Comparison of driving features between ADAS ON and OFF modes (CAN-bus). The four features shown are representative examples selected from a larger set of statistically significant features. The x-axis denotes the driving mode, and the y-axis represents the statistical value of each feature. Asterisks denote statistical significance from an independent *t*-test: *** *p* < 0.001.

**Figure 5:**
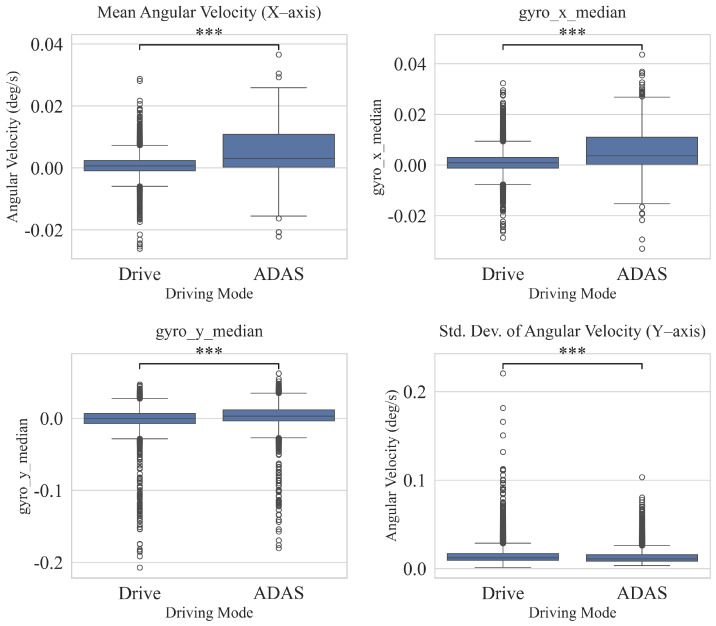
Comparison of driving features between ADAS ON and OFF modes (IMU). Here, gyro_x, gyro_y, and gyro_z denote angular velocities around the roll, pitch, and yaw axes, respectively. Asterisks denote statistical significance from an independent *t*-test: *** *p* < 0.001.

To support qualitative analysis, the distributions of features were visualized using boxplots. As illustrated in Figure 4 and Figure 5, features such as vehicle speed and steering angle exhibit clear differences in median values and variability between ADAS and manual modes, providing an intuitive understanding of behavioral distinctions. Although our analysis identified numerous statistically significant features (*p* < 0.05), those displayed in the figures are a representative subset selected for illustrative purposes. They were chosen to intuitively highlight the core behavioral distinctions found between the two driving modes.

### 5.4. Experimental Results

This section presents the results of ADAS mode classification based on CAN-bus and IMU data, along with behavioral implications. The findings demonstrate that even simple statistical features can effectively distinguish between ADAS-activated and manual driving segments. Notably, pronounced behavioral differences were observed in speed control and steering stability under ADAS activation, while some features remained consistent regardless of ADAS engagement. These results suggest that ADAS operations can be inferred solely from driver behavioral patterns, providing a promising foundation for the development of behavior-based driver monitoring systems.

#### 5.4.1. ADAS ON/OFF Prediction Results

Table 2 presents the performance of the classifier for ADAS mode detection using 4-fold cross-validation. The proposed model achieved an average accuracy of 81.25%, precision of 78.33%, recall of 93.75%, and an F1-score of 83.65%. These results demonstrate that concise statistical and wavelet-based features, combined with leakage prevention and calibrated thresholding, are sufficient to reliably distinguish between ADAS and manual driving segments from CAN-bus data.

Notably, the model exhibited relatively balanced performance across all metrics, indicating that no particular class was favored. This outcome provides a strong baseline for ADAS detection and highlights the potential of in-vehicle sensor data for behavior-based monitoring systems.

Table 3 summarizes the 4-fold cross-validation results of the proposed smartphone IMU-based classifier. The model achieved an average accuracy of 79.18%, precision of 77.90%, recall of 89.12%, and an F1-score of 82.15%. These findings confirm that ADAS activation status can be consistently classified using only a smartphone-based pipeline, without the need for additional hardware.

Furthermore, as shown in Table 4, integrating the CAN-bus and smartphone IMU modalities with the voting-all strategy yielded the highest overall performance among all settings. This result demonstrates the complementary nature of in-vehicle and smartphone signals, suggesting that multimodal fusion provides a more robust solution for reliable ADAS detection.

#### 5.4.2. Comparison Using Raw Data

As shown in Table 5 and Table 6, models trained directly on raw time-series signals suffer from noisy fluctuations and fail to capture the subtle behavioral changes associated with ADAS intervention. In contrast, feature-based pipelines, such as wavelet decomposition with statistical descriptors for CAN-bus data and histogram-based representations for IMU data, emphasize the underlying temporal and frequency-domain patterns. These representations enable the classifiers to form clearer decision boundaries and substantially reduce overfitting and misclassification. These findings confirm that the performance of directly modeling raw signals is limited, underscoring the necessity of applying domain-informed feature extraction prior to classification. Furthermore, this feature-centric approach proved to be highly efficient and practical for real-world deployment. The average prediction time per fold was fast at just 0.27 s for the IMU model and 0.065 s for the CAN-bus model, demonstrating their suitability for real-time applications. The resource requirements were also modest, with peak memory usage of approximately 911 MB for the IMU model and 331 MB for the CAN-bus model, and total training times of 23.4 and 30.3 min, respectively. This combination of robust performance and low computational cost validates the effectiveness of our chosen methodology.

#### 5.4.3. Statistical Analysis of Driving Behavior

The statistical analysis of CAN-bus signals revealed significant differences in 16 out of 21 features between ADAS and manual driving modes (p<0.05). This indicates that the ADAS mode has a statistically substantial impact on overall driving behavior.

Specifically, the mean and median values of speed-related features (C1) were higher in manual driving, suggesting that drivers tended to maintain relatively higher speeds compared to the ADAS mode. In contrast, the mean and standard deviation of steering angle features (C2) were greater in manual driving, indicating more active steering input and higher variability in manual control. These findings imply that ADAS contributes to maintaining a more stable speed and minimizes steering interventions.

However, some torque-related features (C5, C6) did not show statistically significant differences, suggesting that torque control may remain consistent regardless of ADAS intervention under certain driving conditions. Overall, the extended experiment with four drivers confirmed that the ADAS mode stabilizes speed control and reduces steering engagement although some specific signals remained unaffected by the driving mode.

Based on a statistical analysis of IMU signals (accelerometer and gyroscope), significant differences were identified in 15 of 18 features between ADAS and manual driving modes (p<0.05). This suggests that ADAS mode has a statistically significant effect on the vehicle’s linear acceleration (S1) and angular velocity (S4). Specifically, for the accelerometer, the standard deviation on all axes (acc_*std) was lower under ADAS, indicating reduced variability in acceleration, and the lateral (*y*-axis) mean and median were also lower under ADAS, suggesting a suppression of lateral motion. The *x*-axis mean was slightly higher under ADAS, and the median was not significantly different; the *z*-axis mean/median were likewise non-significant (both p>0.05), implying that the central tendency on some axes may not be strongly mode-dependent. For the gyroscope, the mean and median were closer to zero on all axes and the standard deviation decreased (all x/y/z significant), indicating an overall reduction in pitch/roll/yaw variability. Taken together, ADAS appears to smooth vehicle behavior by attenuating small vibrations and rotational fluctuations, whereas the central tendency on certain axes (e.g., acc_x_median, acc_z_mean/median) shows little difference between modes.

## 6. Discussion and Limitations

In this study, synchronized multimodal data comprising IMU sensor and CAN-bus signals were collected during real highway driving. Our analysis showed that steering-related variables exhibited greater variability under manual driving, whereas ADAS activation consistently reduced steering dispersion, indicating stabilized lateral control. Speed-related features also shifted, with manual driving sessions showing higher mean speeds, suggesting that ADAS enforces more conservative cruising. By contrast, torque-related features displayed little or no difference, implying that torque regulation is less sensitive to ADAS engagement. These results highlight that ADAS selectively modulates steering and speed while leaving other dynamics relatively unchanged.

### 6.1. Limitations

The most significant limitation of this study was the small dataset, comprising only four drivers. This small sample size not only raises concerns about the generalizability of our findings but also introduces a considerable risk of model overfitting to the specific behaviors of the participants. While we reported statistical significance using *p*-values, future large-scale studies should complement this by reporting effect sizes (e.g., Cohen’s *d*), confidence intervals, and employing resampling methods such as bootstrapping to provide a more robust assessment of the results.

Furthermore, the scope of the study was constrained by other factors. The current dataset was restricted to vehicle dynamics and driver motion on highways during clear daytime conditions, leaving event-based data and diverse environments underexplored. Highways were prioritized because many production ADAS features are primarily designed for such environments, which allow tighter control of confounding factors. However, this focus limits the applicability of the findings to more complex scenarios. In addition, this study did not analyze the transition periods between ADAS and manual driving, which are critical for understanding driver responses during control shifts.

### 6.2. Future Work

To address these limitations and build upon our findings, we propose several directions for future research. First and foremost, we plan to expand the dataset by recruiting a larger and more diverse pool of participants. This will be crucial for validating our models and ensuring their generalizability.

Second, future work will systematically broaden the data collection to include diverse road contexts (urban, rural, intersections) and environmental conditions (nighttime, rain, fog). This will allow the analysis of ADAS performance and driver behavior in a wider range of real-world scenarios.

Third, we intend to pursue multimodal integration with vision-based data. Incorporating dashcam footage to analyze the external environment or driver-facing cameras to capture gaze patterns, would provide richer context. Fusing vehicle dynamics with visual cues can significantly improve the robustness and interpretability of ADAS status classification.

Finally, a detailed analysis of transition intervals is warranted. Future studies should focus on the hand-off and take-over maneuvers between the driver and system to model the subtle behavioral changes that occur during these critical moments.

Despite these limitations, this work serves as an important proof-of-concept, demonstrating the feasibility of session-level ADAS mode prediction and behavioral evaluation using real-world data. Furthermore, it presents a framework that can be extended toward a comprehensive, scenario-aware ADAS assessment.

## 7. Conclusions

In this study, synchronized IMU sensor and CAN-bus signals were collected during real highway driving to quantitatively analyze the impact of ADAS mode on driver behavior. By comparing driving sessions in which the same drivers traverse identical routes under both ADAS ON and OFF conditions, significant behavioral differences were identified in terms of steering stability, speed control, and torque regulation. Behavior-based classification models further demonstrate that ADAS usage can be successfully inferred solely from sensor data, providing evidence that driver control characteristics undergo substantial changes when ADAS is engaged.

Notably, this study proposed and evaluated an experimental framework based on real multimodal driving data. The results from this framework demonstrate the feasibility of developing ADAS-aware driver monitoring systems and offer a promising starting point for future large-scale validation studies. The findings highlight that behavioral modeling is feasible not only in simulations but also in real-world driving scenarios. Moreover, this work suggests future directions toward personalized, data-driven ADAS design through the integration of additional modalities, such as vision-based data.

## Figures and Tables

**Table 1:** Modalities and representative driving signals included in the dataset.

Category	ID	Signal	Remarks/Unit
CAN-bus signals			
	C1	Vehicle speed	km/h, longitudinal dynamics
	C2	Steering wheel angle	degrees, lateral control input
	C3	Steering rotation velocity	deg/s, steering dynamics
	C4	Actual motor torque	Nm, delivered torque
	C5	Commanded torque	Nm, requested torque
	C6	Rear axle torque	Nm, propulsion load
	C7	Regenerative braking status	On/Off indicator
	C8	Heating system power	W, rear heating element
	C9	Accelerator pedal position	0–100%, driver input
	C10	HV battery current	A, electrical load
	C11	Chiller valve flow	L/min, cooling circuit
	C12	Maximum discharge power	kW, battery safety constraint
	C13	Peak system heating power	W, thermal management
	C14	Pump battery	RPM, cooling pump status
revolutions per minute	C15	Battery voltage	V, electrical system
	C16	System heating power	W, active thermal load
	C17	Rear motor current	A, motor phase current
	C18	Smoothed battery current	A, filtered value
	C19	Requested rear torque	Nm, torque demand
	C20	Rear axle power	kW, delivered power
	C21	Displayed UI speed	km/h, dashboard output
	C22	Minimum cell voltage	V, battery pack
	C23	Maximum cell voltage	V, battery pack
	C24	Axle rotation speed	RPM, wheel dynamics
IMU signals			
	S1	Accelerometer	3-axis linear acceleration (m/s^2^)
	S2	GPS-derived speed	vehicle speed (km/h)
	S3	Orientation	quaternion/Euler angles
	S4	Gyroscope	3-axis angular velocity (deg/s)
	S5	GPS location	latitude/longitude coordinates

ID notation: ‘C’ denotes CAN-bus signals, ‘S’ denotes IMU signals.

**Table 2:** ADAS ON/OFF Prediction Results(CAN-bus).

Method	Accuracy	Precision	Recall	F1 Score
[27]	0.6731	0.7542	0.5417	0.6197
[33]	0.7500	0.8375	0.6875	0.7351
Ours	0.8125	0.7833	0.9375	0.8365

**Table 3:** ADAS ON/OFF prediction results (IMU).

Method	Accuracy	Precision	Recall	F1 Score
[34]	0.6716	0.7500	0.6667	0.6622
[35]	0.5944	0.4702	0.5000	0.4762
Ours	0.7918	0.7790	0.8912	0.8215

**Table 4:** ADAS ON/OFF prediction results (Fusion, Voting-all).

Metric	Accuracy	Precision	Recall	F1 Score
Average	0.8482	0.7671	1.0000	0.8682

**Table 5:** ADAS ON/OFF prediction results (CAN-bus raw data).

Metric	Accuracy	Precision	Recall	F1 Score
Average	0.7500	0.6667	1.0000	0.8000

**Table 6:** ADAS ON/OFF prediction results (IMU raw data).

Metric	Accuracy	Precision	Recall	F1 Score
Average	0.7340	0.7409	0.7701	0.7495

## Data Availability

The data presented in this study are not publicly available due to privacy restrictions.
